# Correction: “When I talk about it, my eyes light up!” Impacts of a national laboratory internship on community college student success

**DOI:** 10.1371/journal.pone.0319821

**Published:** 2025-03-11

**Authors:** Laleh E. Coté, Seth Van Doren, Astrid N. Zamora, Julio Jaramillo Salcido, Esther W. Law, Gabriel Otero Munoz, Aparna Manocha, Colette L. Flood, Anne M. Baranger

The ORCID iDs are missing for the second, fourth, sixth, seventh, eighth and ninth author. Please see the authors’ respective ORCID iDs here:

- Author Seth Van Doren’s ORCID iD is: 0000-0003-0674-277X

(https://orcid.org/0000-0003-0674-277X).

- Author Julio Jaramillo Salcido’s ORCID iD is: 0000-0002-4113-5345

(https://orcid.org/orcid.org/0000-0002-4113-5345)

- Author Gabriel Otero Munoz’s ORCID iD is: 0000-0002-1444-8020

(https://orcid.org/0000-0002-1444-8020)

- Author Aparna Manocha’s ORCID iD is: 0000-0001-7824-9971

(https://orcid.org/0000-0001-7824-9971)

- Author Colette L. Flood’s ORCID iD is: 0000-0002-8674-0872

(https://orcid.org/0000-0002-8674-0872)

- Author Anne M. Baranger’s ORCID iD is: 0000-0002-1973-4632

(https://orcid.org/0000-0002-1973-4632)
