# Supplementary material for: “When I talk about it, my eyes light up!” Impacts of a national laboratory internship on community college student success
Source: PLoS One. 2025 Jan 14;20(1):e0317403. doi: 10.1371/journal.pone.0317403 (PMC11731745; doi:10.1371/journal.pone.0317403)
Supplement: S1 Fig — (PDF) [file pone.0317403.s001.pdf]

S1 Figure. *Community College Internship (CCI) Alumni Survey*, developed for use with CCI alumni.

Questions

First Name

Last Name

If applicable, please provide your previous name(s) used at Berkeley Lab (during your participation in the CCI program).

Contact email address(es)

Please select the term in which you first participated in the CCI program at Berkeley Lab:

If you completed a second CCI term at Berkeley Lab, please select the term:

If you participated in another internship program at Berkeley Lab (e.g., 1st term of SULI), please select the term:

If you participated in additional terms of another internship program at Berkeley Lab (e.g., 2nd term of SULI), please select the term:

Please explain why you initially decided to apply to the CCI program at Berkeley Lab.

If you completed more than one term in any of our internship programs, how did you feel that participating in an additional term would benefit you?

Did you graduate from community college? (Select all that apply)

- ☐ Yes, I obtained an A.A. or A.S. degree (STEM field).
- ☐ No, I'm currently attending community college (majoring in a STEM field).
- ☐ Yes, I obtained an A.A. or A.S. degree (non-STEM field).
- ☐ No, I'm currently attending community college (majoring in a non-STEM field).
- ☐ No, I transferred to a 4-year university without obtaining an A.A. or A.S. degree.
- ☐ No.
- ☐ I decline to state.

Did you attend a 4-year university? (Select all that apply)

- ☐ Yes, I obtained a B.A. or B.S. degree (STEM field).
- ☐ Yes, I attended a 4-year university (majoring in a STEM field), but did not graduate.
- ☐ Yes, I'm currently attending a 4-year university (majoring in a STEM field).
- ☐ Yes, I obtained a B.A. or B.S. degree (non-STEM field).
- ☐ Yes, I attended a 4-year university (majoring in a non-STEM field), but did not graduate.
- ☐ Yes, I'm currently attending a 4-year university (majoring in a non-STEM field).
- ☐ No, I'm currently attending community college.
- ☐ No.
- ☐ I decline to state.

Did you attend graduate school? (Select all that apply)

- ☐ Yes, I obtained an M.A. or M.S. degree (STEM field).
- ☐ Yes, I obtained a Ph.D. degree (STEM field).
- ☐ Yes, I attended graduate school (STEM field), but did not graduate.
- ☐ Yes, I'm currently attending graduate school (STEM field), with the goal of obtaining an M.A. or M.S. degree.
- ☐ Yes, I'm currently attending graduate school (STEM field), with the goal of obtaining a Ph.D. degree.
- ☐ Yes, I obtained an M.A. or M.S. degree (non-STEM field).
- ☐ Yes, I obtained a Ph.D. degree (non-STEM field).
- ☐ Yes, I attended graduate school (non-STEM field), but did not graduate.
- ☐ Yes, I'm currently attending graduate school (non-STEM field), with the goal of obtaining an M.A. or M.S. degree.
- ☐ Yes, I'm currently attending graduate school (non-STEM field), with the goal of obtaining a Ph.D. degree.
- ☐ Yes, I am currently attending graduate school or a professional school not represented by the choices given (e.g., medical school, law school, business school).
- ☐ Yes, I completed graduate-level studies or a professional school not represented by the choices given (e.g., medical school, law school, business school).
- ☐ No, I'm currently attending community college or a 4-year university.
- ☐ No.
- ☐ I decline to state.

Briefly describe your recent educational, professional, extracurricular, and/or personal activities.

Do you feel that your needs as a student were addressed and/or met by participating in the CCI program at Berkeley Lab? Why, or why not?

Briefly describe your recent educational, professional, extracurricular, and/or personal activities.

Briefly describe your "dream job", and why you would like to engage in that type of work.

Please specify in which of the following fields you have been employed, or have professional experience.

In the future, in which of the following fields are you interested in working? (Select all that apply)

- ☐ Academia
- ☐ Industry
- ☐ Government
- ☐ Research
- ☐ STEM policy
- ☐ STEM field, which requires use and/or knowledge of technical skills
- ☐ STEM field, which does not require use and/or knowledge of technical skills
- ☐ U.S. Department of Energy national laboratory
- ☐ U.S. Department of Energy facility (not a national laboratory)
- ☐ STEM education and/or outreach
- ☐ Science media and/or communication
- ☐ Non-STEM field, which requires use and/or knowledge or technical skills
- ☐ Non-STEM field

- ☐ None of these

Since participating in the CCI program, in what capacity have you worked at Berkeley Lab? (Select all that apply)

- ☐ Intern in another program
- ☐ Undergraduate researcher
- ☐ Post-baccalaureate researcher
- ☐ Research associate or research assistant
- ☐ Graduate student researcher
- ☐ Post-doctoral researcher
- ☐ Employee
- ☐ Technical staff
- ☐ None of these

In the future, in which ways are you interested in working at Berkeley Lab? (Select all that apply)

- ☐ Intern in another program
- ☐ Undergraduate researcher
- ☐ Post-baccalaureate researcher
- ☐ Research associate or research assistant
- ☐ Graduate student researcher
- ☐ Post-doctoral researcher
- ☐ Employee
- ☐ Technical staff
- ☐ None of these

Since participating in the CCI program, in what capacity have you worked at any U.S. Department of Energy national laboratory or facility? (Select all that apply)

- ☐ Intern in another program
- ☐ Undergraduate researcher
- ☐ Post-baccalaureate researcher
- ☐ Research associate or research assistant
- ☐ Graduate student researcher
- ☐ Post-doctoral researcher
- ☐ Employee
- ☐ Technical staff
- ☐ None of these

What factors do you believe are important for having a successful research experience at Berkeley Lab?

In your opinion, what makes the undergraduate research experiences at Berkeley Lab different or unique from other internships?

Are you still in touch with any of the members of your Mentor Group? If so, in what capacity?

As a result of your work during the CCI program at Berkeley Lab, please list any related project outcomes, such as publications, presentations, additional collaborations, employment opportunities, etc.

How did your experiences at Berkeley Lab influence your academic or career plans?

If possible, please elaborate on how these experiences did or did not change your perspectives about what it is like to work in research and/or science.

Which activities or experiences from the CCI program made the biggest impact on your career, and why?

Please describe (or give examples) of ways in which you feel that engaging in undergraduate research experiences at Berkeley Lab prepared you to solve problems on other projects, or at other organizations.

What were the most valuable aspects of your experience in the CCI program, and why?

What are ways that the program could be improved to better support community college students?

Please share any memorable stories from your experiences in the CCI program.

These may be experiences which influenced your career goals, had a personal impact on you, changed your perspectives about working in a research-based environment, left you feeling empowered or frustrated, or challenged you in some way. Anything that comes to mind is fair game, and you are encouraged to share as much or as little as you feel comfortable with. Please note that any personally identifiable information about you or others (including names of individuals and group names) will be made anonymous.

Please share any times when you, as an undergraduate, felt like a scientist.
(Or, researcher, biologist, chemist, physicist, mathematician, computer scientist, engineer, etc.)

Please share any other comments you might have about your experiences in the CCI program or working at Berkeley Lab.

What do you think would be an effective way to encourage community college students to apply to the CCI program at Berkeley Lab, or any undergraduate research experience?
