# Supplementary material for: “When I talk about it, my eyes light up!” Impacts of a national laboratory internship on community college student success
Source: PLoS One. 2025 Jan 14;20(1):e0317403. doi: 10.1371/journal.pone.0317403 (PMC11731745; doi:10.1371/journal.pone.0317403)
Supplement: S2 Fig — (PDF) [file pone.0317403.s002.pdf]

S2 Figure. Semi-structured interview protocol, developed for use with CCI alumni.

Questions

Section A.
Tell me a bit about you. What is your name, where are you from, and where do you work or go to school?

Why did you attend college?

What did you major in?

How did you become interested in this topic?

Can you describe what it felt like to be an undergraduate student, just beginning to study science or engineering? (use their field of study)

- How confident were you in your general research or technical skills?
- How confident were you in your ability to succeed in graduate school?
- How confident were you in your ability to succeed in the STEM workforce?

What words would you use to describe your identity?

How does <your field of study> fit in there?

Before becoming involved in research as an undergrad, did you ever feel like a scientist or engineer? (use term they chose)

What are you good at, that makes you well-suited for <their field of study>?

In <their field of study>, who are the people you identify with?

Section B.
Let's talk about the CCI program at Berkeley Lab now.

What happened that made you want to apply to the program in the first place?

Can you briefly describe the type of research you worked on?

What was the benefit of working on-site at Berkeley Lab, instead of collaborating with your team remotely?

Can you share any stories with me about times when you felt successful, as an intern?

What about times when you might have felt unsuccessful?

During the program, how much autonomy did you have as an intern?

How much did you collaborate with others on your CCI project?

When you were an intern, what kinds of conversations would you typically have with your mentors?

In what ways do you feel that participation in this program impacted you personally?

Were there times during the program when you really felt like a scientist or engineer? (use term they chose)

After you completed the CCI program:

- How confident were you in your general research or technical skills?
- How confident were you in your ability to succeed in graduate school?
- How confident were you in your ability to succeed in the STEM workforce?

Section C.
We're almost done! During the next few questions, we will discuss your future.

What are your future academic or career goals?

Thinking now about everything we've discussed, what aspects of your college experience really impacted how you might go about achieving those goals?

Let's pretend for a moment that you have a sibling who is 5-6 years younger than you. Inspired by your career path, your sibling enrolls in the same community college you attended, and declares the same exact major. What advice would you give them, or what strategies would you recommend to them, to support their success in this field?

Can you share any other experiences that you felt were important, that we haven't already discussed?
