# Supplementary material for: “When I talk about it, my eyes light up!” Impacts of a national laboratory internship on community college student success
Source: PLoS One. 2025 Jan 14;20(1):e0317403. doi: 10.1371/journal.pone.0317403 (PMC11731745; doi:10.1371/journal.pone.0317403)
Supplement: S1 Table — The characteristics of each individual are described by more than one category. a These individuals obtained a degree in a non-STEM subject and entered the non-STEM workforce before re-entering school to take STEM coursework at a community college. (PDF) [file pone.0317403.s003.pdf]

S1 Table. Self-reported characteristics of CCI alumni interviewed for this study.

Characteristic		interview subjects	
		n	%
STEM field of study			
	Civil and/or mechanical engineering	4	33
	Physics and/or mathematics	3	25
	Chemistry	2	17
	Biology	2	17
	Environmental Science	1	8
School location			
	Attended a California community college	10	83
	Attended a community college outside of California	2	17
Academic achievement			
	Has a B.A./B.S. in STEM	11	92
	Has an M.A./M.S. in STEM	5	42
	Has a Ph.D. in STEM	2	17
	Studied STEM after obtaining a non-STEM B.A./B.S.[a]	2	17
	Has an advanced degree in health (Ph.D., M.D., D.D.S.)	1	8
Current academic or professional activity			
	Studying/working in a STEM field	11	92
	Working at a DOE national laboratory	3	25
	Attending graduate school for a STEM Ph.D.	3	25

	Studying/working in a health field	1	8
STEM perspectives			
	First in family to study science	8	67
	Perceptions and experiences in STEM impacted by background, culture, and/or identity	6	50
	Passionate about STEM education and outreach	6	50
	"Always" liked science	5	42
	Believes STEM is a pathway to upward mobility	4	33
	Interested in how philosophy and science intersect	3	25
	Became interested in STEM in high school	2	17
Other self-identified characteristics			
	From a low-income family	5	42
	Non-traditional age (during undergraduate studies)	4	33
	First-generation to college	4	33
	Working-class	3	25
	Parent	3	25
	From a rural community	2	17
	Immigrant	1	8
	Child of immigrants	1	8

The characteristics of each individual are described by more than one category. [a] These individuals obtained a degree in a non-STEM subject and entered the non-STEM workforce before re-entering school to take STEM coursework at a community college.
