# Supplementary material for: “When I talk about it, my eyes light up!” Impacts of a national laboratory internship on community college student success
Source: PLoS One. 2025 Jan 14;20(1):e0317403. doi: 10.1371/journal.pone.0317403 (PMC11731745; doi:10.1371/journal.pone.0317403)
Supplement: S2 Table — During interviews, we asked the following two questions: As an undergraduate (before CCI), how confident were you in your general research or technical skills? After you completed the CCI program, how confident were you in your general research skills? These are a selection of the responses we received from CCI alumni, which are representative of the individuals we interviewed (n = 12). Each row contains two quotes, and these are both from the same individual. (PDF) [file pone.0317403.s004.pdf]

S2 Table. Interview responses from CCI alumni about confidence in being successful in the STEM workforce.

Before CCI, how confident were you in your ability to succeed in the STEM workforce?	After CCI, how confident were you in your ability to succeed in the STEM workforce?
"... in terms of getting into a science career, I felt a low sense of confidence before CCI."	"I felt that was much more of a possibility than ever before."
"I wasn't thinking about it, it was more like, 'I'm going to college and then I'm getting a job.' I didn't think about what."	"I think so, because now I had an idea of something I wanted to do in the future. Since I had a crack at it, it was … something I [could] see myself doing and feel confident doing."
"Pretty low. Yeah, I didn't have much confidence. I thought I'd just do physics as a hobby."	"Again, I wouldn't say extremely confident, but from low to mildly confident."
"Yeah, yeah, I think I was. That's why I wanted to have an engineering degree. I feel like it was very straight-forward. I know how to do this skill, and there's a job I could fulfill that requires that skill."	"... it wasn't like I hadn't had experience in the job workforce before. Talking to well-educated academics, people of that different kind of caliber, … I think that was more of an experience for me."
"Not particularly."	"More confident. It [showed] me a different aspect of the workforce where, I think, I felt I excelled more."

During interviews, we asked the following two questions: As an undergraduate (before CCI), how confident were you in your general research or technical skills? After you completed the CCI program, how confident were you in your general research skills? These are a selection of the responses we received from CCI alumni, which are representative of the individuals we interviewed (n=12). Each row contains two quotes, and these are both from the same individual.
