# Supplementary material for: “When I talk about it, my eyes light up!” Impacts of a national laboratory internship on community college student success
Source: PLoS One. 2025 Jan 14;20(1):e0317403. doi: 10.1371/journal.pone.0317403 (PMC11731745; doi:10.1371/journal.pone.0317403)
Supplement: S3 Table — During interviews, we asked the following two questions: As an undergraduate (before CCI), how confident were you in your general research or technical skills? After you completed the CCI program, how confident were you in your general research or technical skills? These are a selection of the responses we received from CCI alumni, which are representative of the individuals we interviewed (n = 12). Each row contains two quotes that are from the same individual. (PDF) [file pone.0317403.s005.pdf]

S3 Table. Interview responses from CCI alumni about confidence in their general research or technical skills.

Before CCI, how confident were you in your general research or technical skills?	After CCI, how confident were you in your general research or technical skills?
“Not very. When I first started community college the thought of doing research was ... I didn’t even understand that research was something you do in an academic setting.”	“I felt stronger ... I don’t know if I considered myself like a great researcher, but I remember at the Lab we did, there was this whole component with CCI where we were doing … some sort of report ... and I came up with this whole research thesis ...”
“No. I was just like, ‘books, study, books, study, books, study.’ ”	“After I got done I was like, ‘Oh, yeah, I can definitely do something else research-wise,’ and now I can actually probably start my data collection and my analysis with whatever tools I needed. Yeah, I definitely have some understanding of how I would set up to study something.”
"I couldn’t grasp what scientists do. I could understand that chemists wear lab coats and do titrations. So, basically, I had no understanding."	“I felt you know, I could do research. Again, I don’t remember one moment of clarity, like, ‘I know this!’ But, it was a gradual process. It was high, I’d say.”
“I had no experience with general research.”	“[The] reality was, that I wasn’t that confident in my lab skills, but I really felt like I’d come a long way in putting together that research paper. I think just the process of writing a paper and doing a poster presentation and going from researching this background of this field and connecting that with my own research, ... it felt like I had gained a lot. For sure.”
“At the time, I didn’t know what research was.”	“Very confident. Now I knew more of what that entails ... when it comes to doing research in biology and that kind of field, when it comes to wet lab stuff, way more confident.”

During interviews, we asked the following two questions: As an undergraduate (before CCI), how confident were you in your general research or technical skills? After you completed the CCI program, how confident were you in your general research or technical skills? These are a selection of the responses we received from CCI alumni, which are representative of the individuals we interviewed (n=12). Each row contains two quotes that are from the same individual.
