# Supplementary material for: “When I talk about it, my eyes light up!” Impacts of a national laboratory internship on community college student success
Source: PLoS One. 2025 Jan 14;20(1):e0317403. doi: 10.1371/journal.pone.0317403 (PMC11731745; doi:10.1371/journal.pone.0317403)
Supplement: S4 Table — During interviews, we asked the following two questions: As an undergraduate (before CCI), how confident were you in your ability to succeed in graduate school? After you completed the CCI program, how confident were you in your ability to succeed in graduate school? These are a selection of the responses we received from CCI alumni, which are representative of the individuals we interviewed (n = 12). Each row contains two quotes that are from the same individual. (PDF) [file pone.0317403.s006.pdf]

S4 Table. Interview responses from CCI alumni about confidence in being successful in graduate school.

Before CCI, how confident were you in your ability to succeed in grad school?	After CCI, how confident were you in your ability to succeed in grad school?
“I didn’t put thought into that. It was, I knew college was a thing I should do, but I didn’t think past that.”	“At that point, I was actually thinking about that more. I was confident enough that was what I wanted to do. I could do it.”
“... it was on my radar, but I didn’t really. I needed to know exactly what I was going to end up focusing on in civil [engineering]. … I don’t think I had thought of research as something I wanted to focus on."	“Well yeah, [CCI] was like grad school 101. That was like, ‘okay, this is a little tiny version of what you have to do in grad school.’ So, yeah, for sure, that was … I don’t know how much closer you can get in an internship with how he worked with us, you know.”
“Oh yeah, no. I didn’t think I could handle community college! Like, grad school was something other people did. No.”	“Definitely was thinking about it. I felt pretty confident that I could get to grad school. I had a better understanding of what that entailed, and what that looked like. Like I said before, we were joking about how we were all going to get our PhDs. Now that was an option.”
“I might’ve had an inflated sense of confidence because I had no idea what it would be like. I was so out of touch, I didn’t really know what a PhD was.”	“I felt excited and optimistic about my ability to [succeed] in grad school afterwards.”
"No, I thought that would be a dead end. Yeah, so I, in the back of my mind, I thought I’d do engineering, so at least I could get a job ... That sounded the most financially viable path for me at the time."	"After research, after [the] CCI program, I said, ‘absolutely I will go for graduate school.’ I think the feeling that I could do research, because there was always a lack of confidence. I thought I was average, and how could I contribute to research? But, going in and doing research, I felt I could make a contribution. That all fell nicely in front [of] me."

During interviews, we asked the following two questions: As an undergraduate (before CCI), how confident were you in your ability to succeed in graduate school? After you completed the CCI program, how confident were you in your ability to succeed in graduate school? These are a selection of the responses we received from CCI alumni, which are representative of the individuals we interviewed (n=12). Each row contains two quotes that are from the same individual.
