# Supplementary material for: “When I talk about it, my eyes light up!” Impacts of a national laboratory internship on community college student success
Source: PLoS One. 2025 Jan 14;20(1):e0317403. doi: 10.1371/journal.pone.0317403 (PMC11731745; doi:10.1371/journal.pone.0317403)
Supplement: S5 Table — In the survey we asked CCI alumni to respond to the following prompt: Briefly describe your “dream job,” and why you would like to engage in that type of work. These are a representative selection of the responses we received. (PDF) [file pone.0317403.s007.pdf]

S5 Table. Survey responses from CCI alumni about their "dream jobs."

Describe your "dream job"
"I want to be a technical manager. I would have to do both technical and managerial work. Also, it would be at a company that cares about having a good workplace culture and investing in its employees."
"I would ideally like to be a research scientist at a DOE National Lab … studying nuclear reactions as they pertain to nuclear astrophysics. I am interested in understanding the origins of the elements in our universe, which many government and university labs are working towards."
"I want to be a research scientist working in a collaboration on a large experiment. I enjoy working with diverse groups of people and I would enjoy … choosing what direction I want my research to go in."
"I would like to do the mechanical work that can best maximize efficiency [for] major utilities … figuring out what would be the best equipment to transport water from treatment plants, or designing the best route for electricity to travel with the least amount of power loss."
"I would like to work as a pharmaceutical liaison acting as a medical and scientific expert engaged in driving key initiatives in research, publications, medical education and field intelligence ... collaborating with researchers and physicians to develop new life saving drugs and treatments."
"My dream job was to be a renewable energy scientist at [DOE national lab] or similar … because I am deeply passionate about the intellectual challenges and excitement of working with cutting edge people."
"My dream job would be to be a design engineer or process engineer for companies such as [for-profit company] or [for-profit company] … because the work of an engineer is directly related to the advances we see in this world today. It is always exciting to say that 'I have been a part of this great invention.' "
"I'd like to work in computational research focusing on the ocean and atmosphere. Computer programming keeps me excited to solve problems every day, and applying it to natural science keeps me interested and passionate about my work on longer time scales."

In the survey we asked CCI alumni to respond to the following prompt: Briefly describe your "dream job," and why you would like to engage in that type of work. These are a representative selection of the responses we received.
