# Supplementary material for: “When I talk about it, my eyes light up!” Impacts of a national laboratory internship on community college student success
Source: PLoS One. 2025 Jan 14;20(1):e0317403. doi: 10.1371/journal.pone.0317403 (PMC11731745; doi:10.1371/journal.pone.0317403)
Supplement: S6 Table — (PDF) [file pone.0317403.s008.pdf]

S6 Table. Coding categories, codes, and sub-codes applied to survey and interview data.

Category from SCCT model	Coding category	Generated codes	Generated sub-codes
Personal inputs	Personal inputs	Gender	
		Race/ethnicity	
		First-generation to college	
Background contextual influences	Background contextual influences	Pre-program social supports and barriers	Attitudes toward community college
			Support from family and friends (of STEM academic/career goals)
Proximal contextual influences	Proximal contextual influences	Pre-program social supports and barriers	Support from people associated with the community college
			Learning experiences available to community college students
		Social supports and barriers	Support from people associated with the learning experience
			Established network associated with the learning experience
			Kindness from people associated with the learning experience
			Mentoring received during the learning experience
Learning experiences	Learning experiences	Pre-program learning experiences (Courses, clubs, other opportunities to learn about STEM)	
		Learning experience (Community College Internship at LBNL)	
	Skill development	Pre-program STEM skills	
		STEM skills	
	Knowledge about STEM careers	Knowledge about STEM careers	

Self-efficacy	Self-efficacy, confidence, STEM identity	Self-efficacy	
		Confidence	
		Feeling like a scientist or engineer (STEM identity)	
Outcome expectations	Outcome expectations	Pre-program STEM outcome expectations	Expectations of admittance into the learning experience (CCI)
			Expectations of success in a STEM career
			Expectations about working in research
			Expectations of graduating from a baccalaureate granting institution
			Expectations of attending graduate school
		STEM outcome expectations	Expectations of success in a STEM career
			Expectations about working in research
			Expectations of graduating from a baccalaureate granting institution
			Expectations of attending graduate school
Interests	Academic and career interests	Pre-program STEM interests	
		STEM interests	Interest in a specific research or STEM field/topic
Choice Goals	Academic and career goals	Academic and career goals	
Choice Actions	Actions	Academic and career actions	
Persistence	Persistence	Persistence in STEM	
